# Localization of acute pyelonephritis in pyrexia of unknown origin using FDG PET/CT

**DOI:** 10.22038/aojnmb.2019.14242

**Published:** 2020

**Authors:** Shrikant Solav, Shailendra Savale, Abhijit Mahaveer Patil

**Affiliations:** SPECT Lab, Nuclear Medicine Services, Pune, Maharashtra, India

**Keywords:** Pyelonephritis, PUO, ^ 18^F-FDG PET CT scan, ^ 99m^Tc-DMSA

## Abstract

**Objective(s):**

Acute pyelonephritis presents with high-grade fever, dysuria, flank pain, leukocytosis, and microscopic hematuria. Urine culture aids in the diagnosis of this infection. It can be complicated or uncomplicated. Complicated pyelonephritis includes uncontrolled diabetes, transplant, pregnancy, acute or chronic renal failure, structural abnormality of the urinary tract, immunocompromised state, and hospital-acquired infections. Gram-negative bacteria commonly involved are *Escherichia, Klebsiella, Proteus, *and *Enterobacter*. The FDG PET/CT helps detect occult causes of fever, such as skeletal tuberculosis, thyroiditis, and lymphoma, when other investigations are inconclusive. We present three cases of pyrexia of unknown origin (PUO) in whom FDG PET/CT helped localize the focus of infection in the kidneys.

**Methods:**

The ^18^F-FDG PET/CT was performed on all three cases and images were acquired using the Biograph Horizon PET/CT system.

**Results:**

A cortical-based focus of FDG uptake was localized in the kidneys. The focus of abnormality was persistent following diuretic administration at 1-hour delayed regional image. Two cases had supportive evidence of pyelonephritis on DMSA scan. One case also had frank pus drained after DJ stenting of the affected side. All of them responded to treatment.

**Conclusion:**

Physiologic excretion of FDG in the urinary tract may interfere with the detection of the focus of infection in the kidneys on FDG PET/CT. However, occult infection in the kidneys may be detected with adequate precautions, such as the use of diuretics and delayed imaging, as illustrated in this case report. Routine investigations were noncontributory in all three cases presenting with PUO. However, FDG PET provided a diagnostic clue for pyelonephritis.

## Introduction

 Acute pyelonephritis presents with high-grade fever, dysuria, flank pain, leukocytosis, and microscopic hematuria. Urine culture aids in the diagnosis of this infection, which can be complicated or uncomplicated. Complicated pyelonephritis includes uncontrolled diabetes, transplant, pregnancy, acute or chronic renal failure, structural abnormality of the urinary tract, immunocompromised state, and hospital-acquired infections. Gram-negative bacteria commonly involved are *Escherichia, Klebsiella, Proteus, *and* Enterobacter*.

 The FDG PET scan is an important investigation to detect the cause of pyrexia of unknown origin (PUO). It helps detect skeletal tuberculosis, thyroiditis, and lymphoma when other investigations are inconclusive. Physiologic excretion of FDG in the urinary tract may interfere with the detection of abnormality in the kidneys in FDG PET/CT. However, occult infection in the kidneys may be detected with adequate precautions, such as the use of diuretics and delayed imaging as illustrated here. Herein, we presented three cases of PUO in whom all routine investigations were noncontributory, and FDG PET provided diagnostic clues for pyelonephritis. 

## Methods

 All cases underwent fluorine-18 FDG PET/CT using 8-10 mCi ^18^F-FDG after a 6-hour fasting state. Whole-body scan was performed using the Siemens Biograph Horizon PET/CT scanner. Images were reconstructed in standard views and interpreted by three experts. Frusemide was administered at a dose of 40 mg IV, and delayed images of the abdomen were acquired over 1 h in all the cases.

***Case 1***

 A 54-year-old man had renal transplantation for end-stage renal disease. He developed renal insufficiency during the 5-month follow-up. On examination, BP was 160/90 mmHg, and creatinine level was 2.4 mg/dl. Ultrasound revealed moderate hydronephrosis and hydroureter. Diuretic renal scan was performed using ethylene dicysteine (EC). Three mCi (111MBq) of Tc-99m-EC was used to acquire sequential static images on the Siemens E.CAM gamma camera with a low-energy high-resolution collimator. 

 Perfusion images at the rate of 2 sec per frame were normal. Sequential static images at the rate of 1 min per frame acquisition showed delayed radiotracer extraction by the transplanted kidney. The intrarenal transit was prolonged. There was progressive radiotracer accumulation in the pelvicalyceal system of the transplanted kidney that did not drain in response to frusemide (1 mg per kg body weight). 

 In view of an obstructed ureter, a pelviureteric diversion procedure was performed (the pelvis of the transplant was anastomosed with the native right ureter). Post-procedure creatinine was 1.2 mg/dl. Four weeks following the surgery, the patient developed fever. Blood culture was sterile. Pseudomonas was isolated in urine culture. Fever continued despite antibiotics. Hence, an F-18-FDG PET/CT scan was performed. The scan showed a focus of increased radiotracer uptake in the inferior pole of the native kidney. The transplanted kidney did not show any abnormal focus of FDG uptake. 

**Figure 1:**
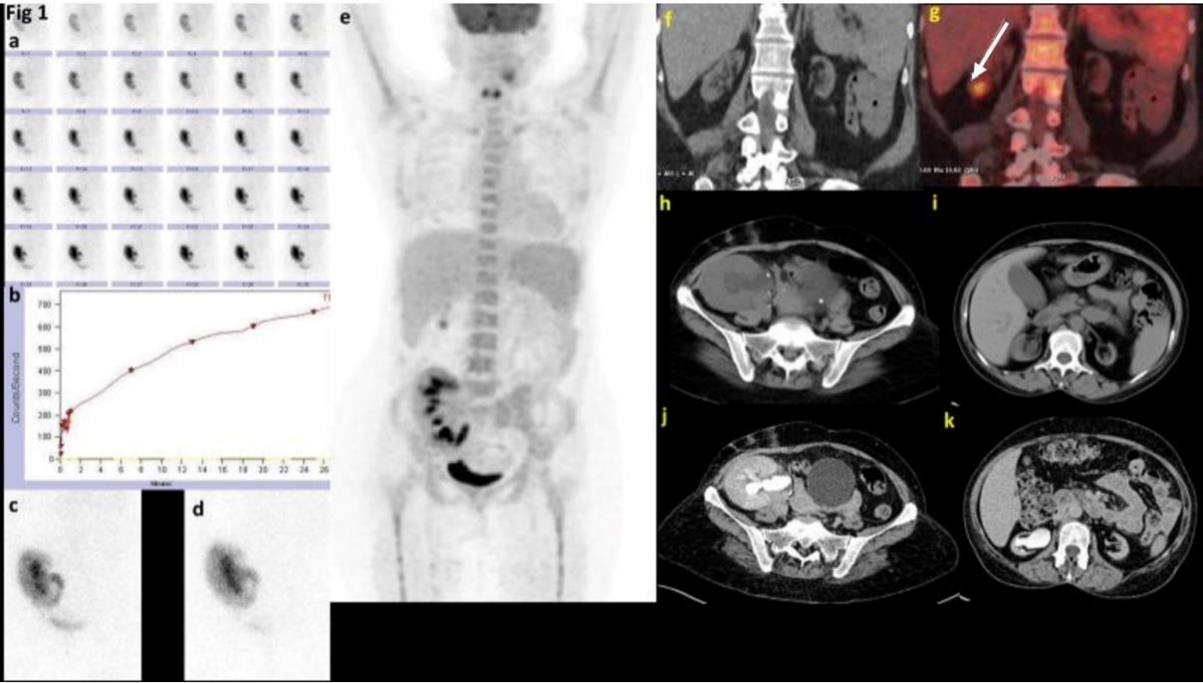
A 54-year-old man post-transplantation developing hydronephrosis in sonography (Renal scan using Tc-99m-ethylene dicysteine shows satisfactory radiotracer uptake that did not clear after Lasix). Renogram pattern was rising [a, b]. Post-void and delayed images showed significant radiotracer retention suggesting obstruction. After pyeloureterostomy, there was fever for which he had F-18-FDG PET/CT scan. It showed a hypermetabolic focus in the inferior pole of the right native kidney [g]. The CT urography showed a normal excretion of contrast from the transplanted kidney [h, j]. The right native kidney showed contrast in the delayed image suggesting reflux [k]. Note that the left native kidney shows no contrast in delayed image.)

 Subsequent CT urography revealed the evidence of reflux from the transplanted renal pelvis to the native ureter (Figure 1). 

There was a disproportionate increase in contrast accumulation in the right native kidney that was not expected in the end-stage renal disease with functioning transplanted kidney. Thus, reflux was considered as the cause of acute complicated pyelonephritis involving the native kidney in this patient. The ^99m^Tc-DMSA scan was not performed in view of the end-stage renal disease. The patient was treated with intravenous antibiotics for 4 weeks, followed by oral antibiotic prophylaxis for 3 months. Her fever resolved with intravenous antibiotics, and her renal function normalized. She was afebrile during the 1-year follow-up.

**Case 2**

 A 65-year-old man had fever and dry cough for 15 days. There was no weight loss or diarrhea. His general examination showed pallor and pedal edema. Respiratory system revealed rales in bilateral lung bases. He had a hemoglobin level of 8.6 gm/dl, white blood cell (WBC) count of 17,900/ml, and platelets count of 278,000/ml. The CT showed extensive cystic bronchiectasis in the lower lobes of the bilateral lung and centrilobular emphysema in the upper lobes.

**Figure 2:**
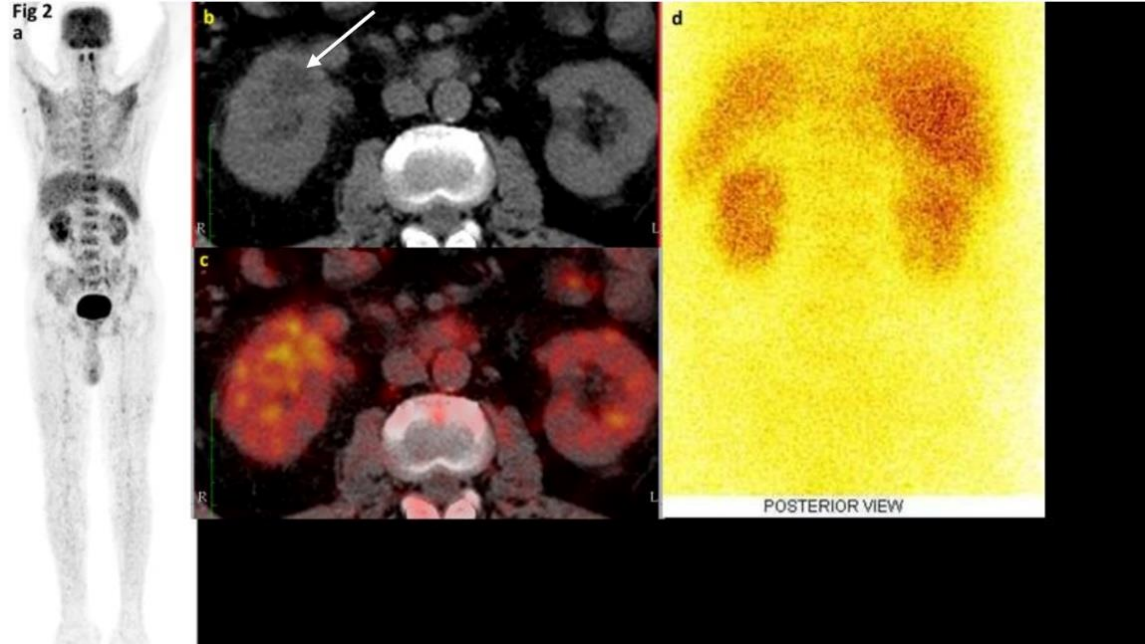
A 65-year-old male with pyrexia undergoing FDG PET/CT for occult infection (Scan showed a focus of hypodensity in the right kidney [b] that showed increased FDG uptake [c]. ^99m^Tc-DMSA scan showed irregular contour of both kidneys suggesting scars [d])

 Urine routine examination was normal. Serum creatinine level was 2.7 mg/dl. The FDG PET scan showed a suspicious metabolically active hypodense focus in the right kidney. Diffusely increased bone marrow uptake was probably marrow reactivation secondary to septicemia. The ^99m^Tc-DMSA renal scan showed subnormal radiotracer extraction by both of the kidneys. The uptake was suboptimal in view of renal dysfunction. However, suspicious irregular renal contour was noted (Figure 2).

 The patient underwent DJ stent that drained frank pus from the right kidney. Thus, the FDG-avid right renal hypodensity was proven to be pyelonephritis. Fever settled within 24 h of DJ stenting. In addition, leukocytosis settled, and patient was afebrile at discharge.

***Case 3***

 A 34-year-old man had a high-grade intermittent fever for several weeks. Clinically, there was mild splenomegaly. He had a hemoglobin level of 12.3 gm%, WBC count of 11000/cmm, ESR rate of 47 mm/h, total bilirubin level of 1.5 mg% (0-1), direct bilirubin level of 1.1 mg% (0.2-1.0), SGOT of 42 IU/L (5-40), SGPT of 36 IU/L (5-35), blood urea of 31.17 mg/dl (20-40), and serum creatinine level of 1.1 mg/dl (0.5-1.5). Rapid malaria parasite test was negative.

 Widal test for enteric fever was positive, antithyroglobulin antibody test showed a level of <15.0 U/ml (0-60), and Mantoux test was negative. 

 Blood culture was sterile. The Ultrasound showed mild splenomegaly and two 4-mm calculi, each of which was located in the mid pole of the right and left kidneys. No focal lesion was seen in the spleen or liver. 

**Figure 3:**
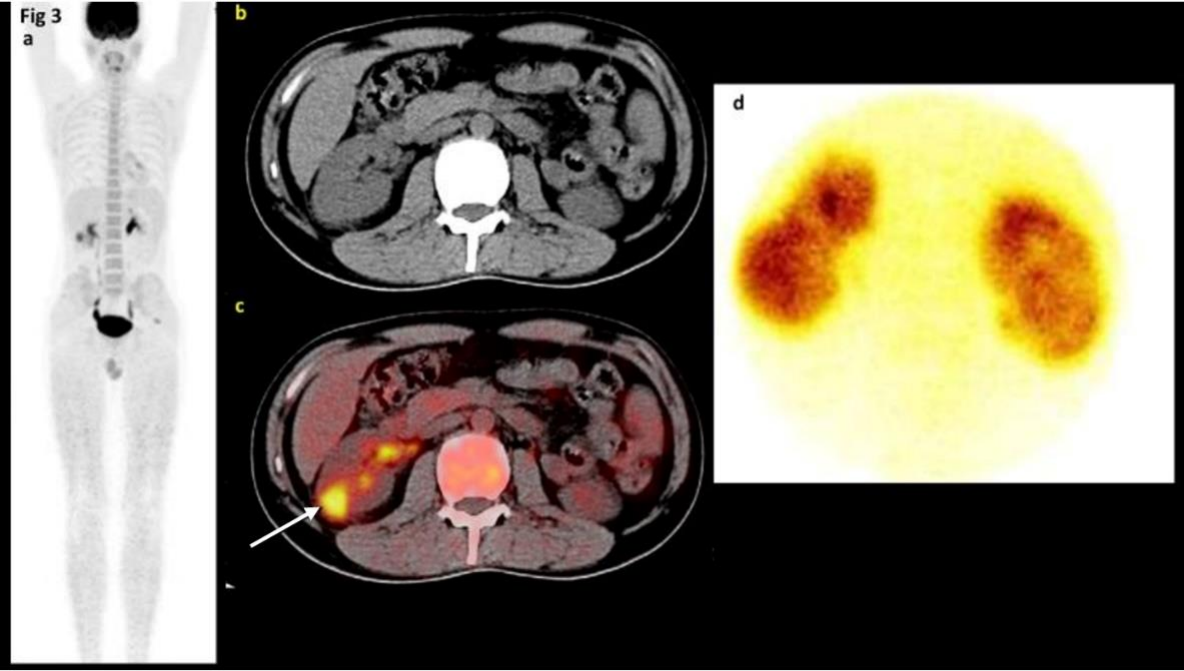
A 38-year-old male with fever and no detectable cause undergoing FDG PET/CT scan (There was a focus of increased FDG uptake in the inferior pole of the right kidney [c]. The ^99m^Tc-DMSA scan showed photon deficiency in the right kidney laterally corresponding to focal FDG uptake compatible with acute pyelonephritis [d]. The left kidney also showed irregular contour suggesting scars.)

 Echocardiogram was normal. Follow-up investigations demonstrated an ESR of 60 mm/h, CRP of 62.78 mg/L, and ACE of 7.6.17:77.32 U/l (8.0-52.0) and 7.6.17 with negative results for *Brucella* IgG/IgM, CCP-Ab, and RA factor. Bone marrow biopsy showed normal trilineage hemopoiesis with no evidence of malignancy or tuberculosis. In addition, the CT scan revealed hepatomegaly and the prominence of the lower part of the right ureter without any obvious obstructive causes at the vesicoureteric junction. The FDG PET/CT revealed a hypermetabolic cortical based focus in the lower pole of the right kidney (Figure 3). Subsequent urine culture revealed *Klebsiella* that responded to antibiotics.

## Discussion

 Focal FDG uptake in acute complicated pyelonephritis has been reported earlier. Wan et al. observed such pattern in 61% of their cases (19 of 31 patients). In this study, they observed that patients with focal uptake had a high incidence of abscess formation requiring drainage. Only one of the three cases reported here had abscess that was drained using DJ stent (1). Focal FDG uptake in a horseshoe kidney secondary to pyelonephritis has also been reported (2).

 Diffuse uptake of FDG in renal parenchymain xanthogranulomatous pyelonephritis has been reported. The term is derived from the replacement of renal parenchyma with lipid-laden macrophages (xanthoma cells) associated with chronic suppuration and destruction. Reportedly, 10% of patients with xanthogranulomatous pyelonephritis are diabetic (3).

 The ultrasound is a readily available test in suspected pyelonephritis and carries low sensitivity in a range of 20-30% (4). Enlarged kidney, loss of renal sinus fat (edema), changes in echogenicity, hypoechoic lesion due to edema, hyperechoic lesion due to hemorrhage, loss of corticomedullary differentiation, and abscess formation are some features of US. Tissue harmonic imaging has increased the sensitivity and specificity to as high as 90% (5).

 The CT provides complete information regarding pyelonephritis. Unenhanced CT detects gas, calculi, hemorrhage, inflammatory masses, and abscess (6). Contrast-enhanced CT shows wedge-shaped areas of poor enhancement from papilla to renal cortex. However, delayed images at 3-6 h show persistent enhancement (7). The CT has the 

advantage of detecting the additional signs of renal infection, such as perinephric fat stranding, thickening of Gerota’s fascia, and abscess formation (8).

 The MR is used where CT is contraindicated (e.g., pregnancy and renal insufficiency). The MR also demonstrates enlarged kidney, edema, hemorrhage, abscess, and perinephric collection. Inflammatory lesions show low signals on T1W and high signals on T2W images. Contrast MR shows wedge-shaped striated areas of decreased enhancement (9).

 The ^99m^Tc-DMSA (dimercaptosuccinate) scan is characterized by cortex-based wedge-shaped defect. Acute pyelonephritis is usually associated with preserved renal volume. Recurrent pyelonephritis may result in the loss of volume with irregular cortical outline. However, it is difficult to differentiate chronic scar from acute pyelonephritis unless it is correlated with other imaging (10). 

 Yoo et al. compared Doppler ultrasound, ^99m^Tc-DMSA, and CT scan in acute pyelonephritis and found that ^99m^Tc-DMSA and CT were equally sensitive in detecting pyelonephritis (11). Our case report highlights the importance of CT in the detection of reflux post-pyeloureteric anastomosis. The FDG PET/CT was reported to detect pyelonephritis in 5 of 112 cases presenting with PUO by Gafter Gvili et al. Four of these cases were renal transplants, and one was native kidney (12). The FDG PET/CT seems to be an unavoidable investigation in the workup of PUO (13).

 Because of the physiologic excretion of radiotracer in the urinary system, occult infection may be masked on FDG PET/CT. Hence, 60-min delayed post-diuretic imaging of the kidneys should be acquired to localize a focus of pyelonephritis as in the presented cases. Sonography, CT, and MR have their own place in the diagnostic algorithm of PUO and detect structural changes. On the other hand, FDG PET detects metabolic changes at a cellular level; therefore, it is a sensitive method to diagnose changes at a molecular level before the manifestation of structural abnormality. 
